# New findings of terrestrial arthropods from the Azorean Islands

**DOI:** 10.3897/BDJ.12.e136391

**Published:** 2024-11-07

**Authors:** Mário Boieiro, Zsófia Varga-Szilay, Ricardo Costa, Luis Crespo, Abrão Leite, Raúl Oliveira, Gabor Pozsgai, Carla Rego, Hugo Renato Calado, Mário Brum Teixeira, David H. Lopes, António Onofre Soares, Paulo A.V. Borges

**Affiliations:** 1 University of the Azores, cE3c- Centre for Ecology, Evolution and Environmental Changes/Azorean Biodiversity Group, CHANGE – Global Change and Sustainability Institute, School of Agricultural and Environmental Sciences, Rua Capitão João d´Ávila, Pico da Urze, 9700-042, Angra do Heroísmo, Azores, Portugal University of the Azores, cE3c- Centre for Ecology, Evolution and Environmental Changes/Azorean Biodiversity Group, CHANGE – Global Change and Sustainability Institute, School of Agricultural and Environmental Sciences, Rua Capitão João d´Ávila, Pico da Urze, 9700-042 Angra do Heroísmo, Azores Portugal; 2 IUCN SSC Atlantic Islands Invertebrate Specialist Group, Angra do Heroísmo, Azores, Portugal IUCN SSC Atlantic Islands Invertebrate Specialist Group Angra do Heroísmo, Azores Portugal; 3 Doctoral School of Biology, Institute of Biology, ELTE Eötvös Loránd University, Budapest, Hungary Doctoral School of Biology, Institute of Biology, ELTE Eötvös Loránd University Budapest Hungary; 4 LIBRe – Laboratory for Integrative Biodiversity Research, Finnish Museum of Natural History, University of Helsinki, Helsinki, Finland LIBRe – Laboratory for Integrative Biodiversity Research, Finnish Museum of Natural History, University of Helsinki Helsinki Finland; 5 Rua Fernando Pessoa, nº99 R/C DTO 2765-483, Estoril, Portugal Rua Fernando Pessoa, nº99 R/C DTO 2765-483 Estoril Portugal; 6 Mestrado em Gestão e Conservação da Natureza, University of the Azores Rua Capitão João d´Ávila, Pico da Urze 9700-042, Angra do Heroísmo, Azores, Portugal Mestrado em Gestão e Conservação da Natureza, University of the Azores Rua Capitão João d´Ávila, Pico da Urze 9700-042 Angra do Heroísmo, Azores Portugal; 7 cE3c- Centre for Ecology, Evolution and Environmental Changes, CHANGE – Global Change and Sustainability Institute, Faculty of Sciences, University of Lisbon, Lisboa, Portugal cE3c- Centre for Ecology, Evolution and Environmental Changes, CHANGE – Global Change and Sustainability Institute, Faculty of Sciences, University of Lisbon Lisboa Portugal; 8 University of the Azores, cE3c- Centre for Ecology, Evolution and Environmental Changes/Azorean Biodiversity Group, CHANGE – Global Change and Sustainability Institute, School of Sciences and Technology, Rua da Mãe de Deus, 9500-321, Ponta Delgada, Azores, Portugal University of the Azores, cE3c- Centre for Ecology, Evolution and Environmental Changes/Azorean Biodiversity Group, CHANGE – Global Change and Sustainability Institute, School of Sciences and Technology, Rua da Mãe de Deus, 9500-321 Ponta Delgada, Azores Portugal; 9 University of the Azores, Biotechnology Centre of Azores, School of Sciences and Technology, Ponta Delgada, Azores, Portugal University of the Azores, Biotechnology Centre of Azores, School of Sciences and Technology Ponta Delgada, Azores Portugal; 10 IUCN SSC Monitoring Specialist Group, Angra do Heroísmo, Azores, Portugal IUCN SSC Monitoring Specialist Group Angra do Heroísmo, Azores Portugal

**Keywords:** Azores, biodiversity conservation, exotic species, island biodiversity, species introductions, species inventory

## Abstract

The knowledge on taxonomic diversity of arthropods is key to better understanding the biodiversity patterns and processes and guiding sustainable conservation strategies and practices. In the Azores, terrestrial arthropods are relatively well-inventoried following the publication of comprehensive checklists that have been regularly updated. Nevertheless, every year, new species are found as a result of new arrivals to the Archipelago and from addressing specific taxonomic lacunae. Here, we update the taxonomic terrestrial arthropod biodiversity of the Azores by reporting for the first time 13 species for the Archipelago, namely *Oligonychusperseae* Tuttle, Baker & Abbatiello, 1976, *Textrixpinicola* Simon, 1875, *Pholcommagibbum* (Westring, 1851), *Schistocercagregaria* (Forsskål, 1775), *Phoracantharecurva* Newman, 1840, *Diachusauratus* Fabricius, 1801 *Phyllotretaprocera* (Redtenbacher, 1849), *Phyllotretastriolata* (Fabricius, 1803), *Diboliaoccultans* (Koch, 1803), *Pseudolynchiacanariensis* (Macquart, 1839), *Hermetiaillucens* (Linnaeus, 1758), *Dryocosmuskuriphilus* Yasumatsu, 1951 and *Ectemniuscephalotes* (Olivier, 1792), and several new species records for specific islands. These species benefitted from the increase in transportation of goods and commodities, both from outside the Archipelago and between islands, to arrive and spread across the Archipelago with some of them posing new challenges to local agriculture, forestry and biodiversity conservation management.

## Introduction

Having detailed and up-to-date knowledge about biodiversity is crucial to better understand the human-biosphere interaction, develop adequate and sustainable management practices and support decision-making in a wide variety of thematics. This issue is particularly important in island ecosystems where many endemic species are threatened by extinction, but also because human societies and human well-being are particularly vulnerable to the severe threats posed by biological invasions to island economies, through impacts on the agriculture, forestry, veterinary, public health, tourism, infrastructure, etc. (Fernández-Palacios et al. 2021). Thus, regular biodiversity monitoring and reporting on its changes may allow the development of conservation strategies supported by scientific evidence and the timely adoption of more effective preventative and control measures (Silva-Rocha et al. 2018).

The Azores Archipelago, located in the Macaronesia Region in the North Atlantic, presents a unique biodiversity with many endemic plants and animals, but has also been subjected to high numbers of species introductions mediated by human activities, particularly commerce and tourism (Borges et al. 2010, Rego et al. 2015, Borges et al. 2022a). Many of these introductions are terrestrial arthropod species that proved to be very harmful to the Azorean ecosystems, economy and local communities (Silva et al. 2008). For example, the introduction of the Japanese beetle *Popilliajaponica* Newman, 1841 (Coleoptera, Scarabaeidae) to Terceira Island led to significant impacts on agriculture since this species feeds on plant roots during the larval stage while the adults are herbivorous on diverse species, including some that are key for the local economy like grapevine, maize, pear and apple trees and several ornamental species (Vieira 2008 and references therein). Following its introduction, the species has spread within and between Azorean islands and the efforts to its eradication were unsuccessful. Data on pest control costs and economic losses caused by the *P.japonica* invasion in the Azores are not available, but estimated to be very high. The establishment and spread of two exotic termites (*Cryptotermesbrevis* (Walker, 1853) and *Reticulitermesgrassei* Holmgren, 1913) in the Azores is also a major problem due to the significant damage caused to the housing system and built heritage in several town centres (mainly Angra do Heroísmo, Horta and Ponta Delgada) (Borges and Myles 2007). The severe impacts of these urban pests led to the creation of specific legislation to set measures for building disinfestations, grant financial support for repair works on properties damaged by termites and establish the regime to be applied to the transport and final destination of infested materials. Simultaneously, multiple monitoring and control/eradication programmes were carried out in the locations affected by termite infestations. The economic costs associated with losses and management of termite invasion are over 226 million euros and are expected to rise in the near future (Guerreiro et al. 2014).

The introduction of exotic species in the Azores is considered one of the major challenges for biodiversity conservation and environmental sustainability, reinforcing the urgency in tackling this issue (Silva et al. 2008, Borges et al. 2020). Since the end of the 20^th^ century, but particularly early this century, there have been joint efforts, including the governmental authorities and academia, to inventory and monitor the biodiversity of the Azores which have led to the publication of a first checklist (Borges et al. 2005) that has been regularly updated (e.g. Borges et al. (2010), Borges et al. (2022a)) and made available online. The availability of this online infrastructure for universal use by researchers, stakeholders, decision-makers and general public (see AZORESBIOPORTAL), has proven to be fundamental for the development of biological conservation programmes and education. The rapid communication of novel taxonomic findings in the Azorean islands, particularly when associated with dynamic species checklists providing identification tools and distribution maps, is paramount to protect native biodiversity and ensure the well-being of local populations. In the last decade, Borges et al. (2013), Soares et al. 2021, Borges et al. (2022) and Boieiro et al. (2023) recorded several new introductions of exotic species, many of them with invasion potential. Here, we report the new arrival of several terrestrial arthropod species in the Azores islands and highlight the need to monitor their spread, particularly for some species that may be considered problematic.

## Materials and methods

The new species records result both from ongoing inventory/monitoring efforts to improve the knowledge on the Azorean terrestrial arthropod biodiversity, but also from occasional sampling. The use of standardised sampling techniques to monitor epigean and canopy arthropods [BALA protocol, following Biodiversity of Arthropods of Laurisilva of Azores (BALA) project], airborne arthropods [Sea Land Aerial Malaise protocol, following Sea, Land, Aerial Malaise (SLAM) project] and insect pollinators [SPRING protocol, following Strengthening Pollinator Recovery through INdicators and monitorinG (SPRING) project] led to the collection of new species findings at island or archipelago level (Borges 2005, Gaspar et al. 2008, Lhoumeau et al. 2022, Pozsgai 2024, see also https://pollinator-monitoring.net/). The BALA protocol combines the use of standardised pitfall trapping using both attractive (Turquin) and passive (ethylene glycol) solutions and standardised beating which was performed on the three most dominant tree species in each study site (e.g. Gaspar et al. (2008)). The SLAM protocol uses sea, land and air Malaise traps to collect flying insects and other arthropods (e.g. ballooning spiders) throughout the year (Lhoumeau et al. 2022). We also applied a modified version of the SPRING protocol combining pan trapping and direct collections along 50 m transect walks. Additionally, occasional sampling, targeting less studied hyperdiverse taxonomic groups (e.g. Diptera, Hymenoptera), allowed us to record species hitherto unknown in the Azores. The specimens were identified to species level in the laboratory using a Leica S9i stereomicroscope and diverse taxonomic literature (see below). All collected specimens are stored in vials with ethanol (70%) and were deposited in the Dalberto Pombo entomological collection (DTP) at the University of the Azores (Angra do Heroísmo, Portugal). All information associated with the new taxonomic findings, namely on the study specimens, sampling techniques and collection sites and dates, follows the Darwin Core standards and is available in GBIF (Boieiro and Borges 2024).

### Study area

The studies were carried out in the Azores Archipelago, which is located in the North Atlantic (Fig. 1). The Archipelago has nine islands distributed amongst three island groups (Fig. 1). The islands differ from each other with regard to geological age, area, altitude, habitat diversity and natural vegetation coverage (Gaspar et al. 2008, Rego et al. 2015). The climate is temperate oceanic with regular and abundant rainfall, particularly during autumn and winter. The landscape is dominated by human-transformed habitats, mainly semi-natural and intensive pastures and forest plantations (dominated by *Pittosporumundulatum* Vent., *Eucalyptusglobulus* Labill., *Cryptomeriajaponica* (L. fil.) D. Don, *Acaciamelanoxylon* R. Br. and *Pinuspinaster* Aiton), while native forests are now restricted to ~ 5% of the total area of the Archipelago (Gaspar et al. 2008, Rego et al. 2015). Nearly 250,000 people live in the Azores, but the human population is very unevenly distributed amongst the islands. Recently, there has been an exponential increase in tourism from different geographic origins that poses significant challenges to the regional authorities and raises concern about the potential impacts on native biodiversity, local economy and human well-being (see https://otacores.com/estatistica/passageiros-desembarcados/).

## Results

Thirteen species are recorded for the first time to the Archipelago, while several other species, previously recorded in the Azores, are now reported to other islands. Most of these species are exotics and their arrival and spread raises concerns due to their potential impacts on local economy, native biodiversity and natural ecological processes. A list of the terrestrial arthropod species is presented below (Table 1), followed by sections with comments on each single species. Detailed information on the study specimens and collection sites is available in GBIF (Boieiro and Borges 2024).

### Oligonychusperseae Tuttle, Baker & Abbatiello, 1976 (Acarina, Tetranychidae)

The Persea mite, *Oligonychusperseae* Tuttle, Baker & Abbatiello, 1976, is primarily known for its association with avocado crops and can be a major pest in avocado orchards (Torres et al. 2024). Heavy infestations can reduce photosynthetic capacity of the host plants, leading to reduced fruit quality and yield (Liburd et al. 2019). The pest can be observed along the veins of the underside of avocato leaves, where it completes its life-cycle (EFSA Panel on Plant Health (PLH) et al. 2022). *Oligonychusperseae* adults measure roughly 0.5 mm length, being often yellowish-green with dark spots on their bodies (Menge and Ploetz 2003). This species is a major pest in the Canary Islands and Madeira, has been spreading into several islands and its invasion led to the development of new integrated pest management strategies for avocado production (Torres et al. 2024). The species was found attacking avocado orchards at Terra-Chã and São Pedro (Terceira, Island). In the Azores, the avocado orchards are scarce and the impacts of this new pest are still to be quantified.

### Textrixpinicola Simon, 1875 (Araneae, Agenelidae)

*Textrixpinicola* Simon, 1875 is a new record for the Azores Archipelago. It was found in Flores Island in a low elevation mixed forest dominated by *Acaciamelanoxylon* within the scope of the project LIFE-BEETLES (Lhoumeau et al. 2024). This species occurs in southern Europe, from mainland Portugal to Italy (World Spider Catalogue 2024). It is commonly found in tree bark, where it builds its funnel-web, although it can also be found on the ground.

### Agynetarugosa Wunderlich, 1992 (Araneae, Linyphiidae)

*Agynetarugosa* Wunderlich, 1992 is an endemic Azorean species previously known from Faial, São Jorge and São Miguel Islands (Borges et al. 2022a). We report a new record in Terceira Island. The species was found at low elevation in a native forest dominated by *Ericaazorica* at Farol da Serreta (Lhoumeau et al. 2022).

### Phidippusaudax (Hentz, 1845) (Araneae, Salticidae)

**Figure 2.**
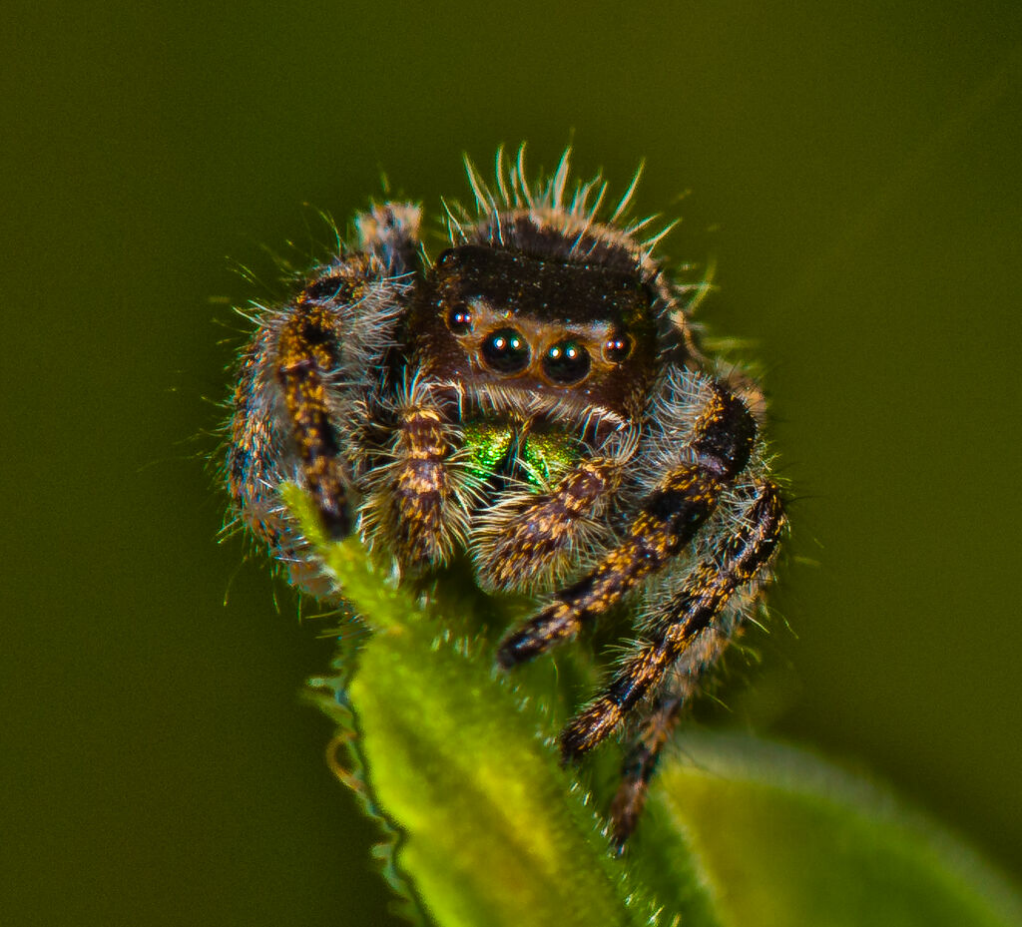
Bold jumping spider *Phidippusaudax* (Hentz, 1845). Photo by Paulo A.V. Borges.

The bold jumping spider, *Phidippusaudax* (Hentz, 1845), is currently spreading in Terceira Island (Fig. 2). Originally found near the airport at Praia da Vitória (Borges et al. 2013), this species in now common in the main town of Angra do Heroísmo. Its origin is North America, ranging from southern Canada to northern Mexico. *Phidippusaudax* is a fascinating species due to its complex behaviour and adaptability to different environments (Bednarski et al. 2012). Its role as a natural pest controller also highlights its ecological importance (Young 1989).

### Dipoenaumbratilis (Simon, 1873) (Araneae, Theridiidae)

*Dipoenaumbratilis* (Simon, 1873), commonly known as the comb-footed spider or cobweb spider, was recently recorded in Faial Island by Lamelas-Lopez et al. (2023), but without mentioning the fact that this was the first record for the Azores. Distribution data on this species for Faial Island can be accessed in GBIF (Borges and Lamelas-López 2023). In addition, the species was also recently found at Mistério de St. Luzia (Pico Island) under the scope of the SLAM project. The species is known from Spain and Portugal (Branco et al. 2019) and it is morphologically characterised by a dark or brownish colouration. The specimens were collected from the vegetation in the Botanical Garden of Faial Island by both beating and direct observations. In Spain, this species has been found in relative high numbers in tree cavities (Barrientos et al. 2022).

### Pholcommagibbum (Westring, 1851) (Araneae, Theridiidae)

*Pholcommagibbum* (Westring, 1851) was recently recorded on Flores Island by Lhoumeau et al. (2022) and Lhoumeau et al. (2024), but without mentioning the fact that this was the first record for the Azores. Distribution data on this species can be accessed in GBIF (Borges and Lhoumeau 2024a, Borges and Lhoumeau 2024b). This typically epigean species has also been found to occur to at least 1 m depth in the soil in Czechia, occupying the whole sampled soil profile (Laška et al. 2011). However, it hunts using a web derived from those typically built by other theridiids such as *Achaearanea* species (Benjamin and Zschokke 2003).

### Zoropsisspinimana (Dufour, 1820) (Araneae, Zoropsidae)

**Figure 3.**
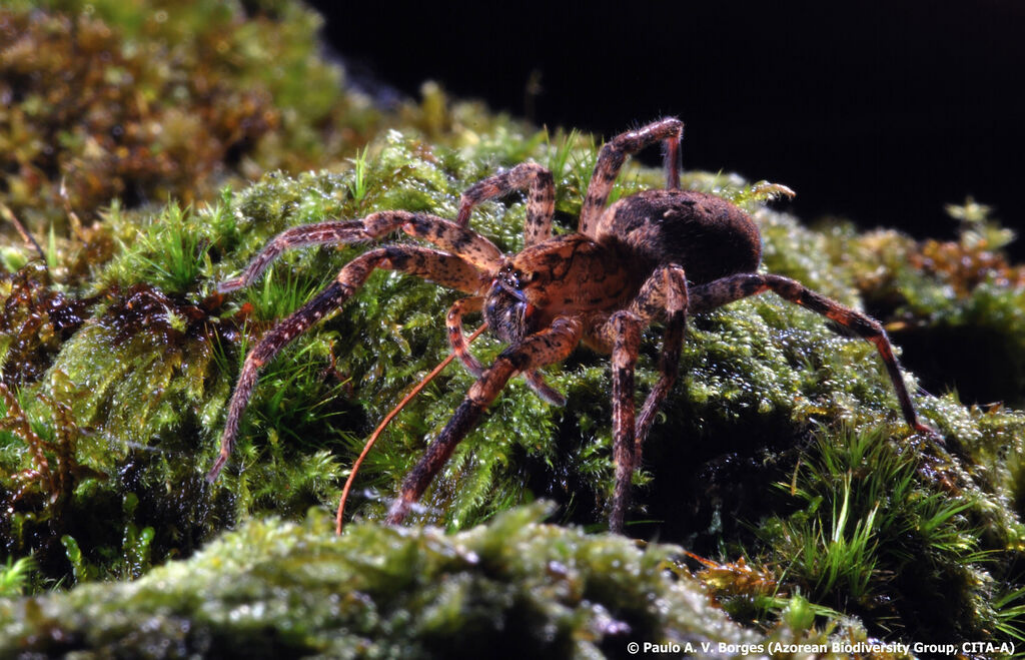
False wolf spider *Zoropsisspinimana* (Dufour, 1820). Photo by Paulo A.V. Borges.

*Zoropsisspinimana* (Dufour, 1820) is commonly known as the false wolf spider (Fig. 3). This is a new record to Flores Island. The specimens were collected in vegetables gardens at low altitude. Previously known from Faial, Pico and Terceira (Borges et al. 2022a), this Mediterranean species is spreading across the Azores (Borges et al. 2013), as well as in the Canary Islands (personal communication by Daniel Ramos), likely expanding its range through human activities, such as trade and transportation. The spread within Europe is facilitated by its adaptability to urban environments and ability to withstand cooler climates (Wittenberg 2005). Although the large size of this species often alarms humans, bites are not of medical importance, being equivalent to a slight sting of a bee (Pospischil 2011). This species may compete with native spiders for resources and also feed on other spiders (Wirth and Schulemann-Maier 2024), potentially displacing them. Given its large size and ease of observation, this species is an excellent model for reporting in citizen-science programmes making it easier to monitor and manage its spread.

### Schistocercagregaria (Forsskål, 1775) (Orthoptera, Acrididae)

The desert locust, *Schistocercagregaria* Forsskål, 1775, is one of the most feared agricultural pests since early civilisation, with plagues documented from West Africa to Southwest Asia. For this reason, this report can be seen as a major putative problem to the islands economy and fauna. In the swarming phase of their life cycle, they can wipe out crops and wild vegetation with incredible voracity and speed (Cressman 2016, Githae and Kuria 2021). The captured individual was photographed and later identified with the use of a taxonomic key (Llucià-Pomares 2002), following an initial identification on iNaturalist. Specimen morphology corresponds to the solitary phase, supporting the hypothesis that it was a lone vagrant that arrived on Terceira Island through the strong winds blowing from North Africa, which were prevalent at the time of capture (22.11.2023). Nonetheless, this species is present in the Canary Islands (Bland et al. 1996, Arechavaleta et al. 2010), mostly as a migrant with some instances of swarms having been observed as in 2004, in Lanzarote (Petit and Prudent 2010). Thus, we cannot disregard the possibility of such record corresponding to an occasional occurrence in the Azores. However, even if this species establishes in the Azores, it is unlikely to go into a gregarious phase as rapid reproduction and an increase in population density would require a period of drought and low availability of food resources followed by heavy rains and plant abundance, which is very unlikely to occur in the Archipelago (Petit and Prudent 2010). The Azores being known for its consistent high humidity and plant cover, would in theory, help to keep populations stable during their solitary phase.

### Trigonidiumcicindeloides Rambur, 1838 (Orthoptera, Trigonidiidae)

The delicate bush cricket *Trigonidiumcicindeloides* Rambur, 1838, was first recorded for S. Miguel Island by Borges et al. (2013). Later, this species was recorded from the coastal wetlands of Praia da Vitória in Terceira Island by Borges et al. (2018). Now it was found for the first time on Santa Maria Island, near the airport, by Nuno Bicudo. Interestingly, this species was found in the vicinity of airports on all the three islands, which suggests that its expansion is likely human-mediated. In other invaded areas, like the Rodrigues Island, in Mascarenes, this species is expanding its range (Hugel 2012). *Trigonidiumcicindeloides* inhabits wetlands, such as lake and river shores and is considered a pest of rice in India (Garg and Tandon 1982), but in the Azores, it seems to be restricted to urban and ruderal areas. Like many crickets, *T.cicindeloides* produces characteristic chirping mating calls (Huber et al. 2019).

### Nabiscapsiformis Germar, 1838 (Hemiptera, Nabidae)

The pale damsel bug, *Nabiscapsiformis* Germar, 1838 is the most widespread species in its genus, being found in North and South America, Russia, Africa and Europe (Péricart 1987, Krey and Renkema 2018). As predators of caterpillars and aphids in all their life stages, this species can be beneficial to farmers and gardeners. Since all species of this genus are predators of small arthropods and may show cannibalistic behaviour when prey is scarce, this newcomer may compete with the native *Nabispseudoferus* Remane, 1949. This species was previously known from other Azorean islands (Borges et al. 2010, Borges et al. 2022a) and is now reported for the first time to Terceira Island.

### Phoracantharecurva Newman, 1840 (Coleoptera, Cerambycidae)

The eucalyptus longhorned borer, *Phoracantharecurva* Newman, 1840 (Fig. 4), is a medium-size beetle (15-30 mm) that is native to Australia and Papua New Guinea, but has been introduced in many countries in Africa, America and Europe (EPPO 2024). This species has been reported from several *Eucalyptus* species and, more rarely, from other host plants (Bybee et al. (2004), see also https://doi.org/10.1079/cabicompendium.40371). It is often considered a pest species since it attacks living trees, particularly if they are stressed by drought or if their stems and branches are dying. This beetle is very similar to *Phoracanthasemipunctata* (Fabricius, 1775), which is also a widely distributed eucalyptus forest pest and with which it may co-occur (see https://doi.org/10.1079/cabicompendium.40372). Contrary to *P.semipunctata*, the adults of *P.recurva* have antennae with dense and long golden hairs, the hind femur has strong spines on antero-dorsal side and the base of elytra is predominantly yellowish, with reduced black spots (Wang 1995). This is the first record of this species for the Azores. A single specimen was found in the Campus of the University of the Azores (Angra do Heroísmo, Terceira), where several individuals of *Eucalyptusglobulus* grow. Since this species is considered a forest pest, the regional forestry services were alerted to the arrival of this exotic species in the Azores so that the necessary measures for its control/monitoring can be taken briefly.

### Diachusauratus Fabricius, 1801 (Coleoptera, Chrysomelidae)

The bronze leaf beetle (Fig. 5) belongs to the case-bearing leaf beetles (Cryptocephalinae), listed in the Cryptocephalina subtribe, implying a close relationship with the Europe-wide common genus of *Cryptocephalus* (Chamorro-Lacayo and Konstantinov 2004). It is widespread in the New World and it most likely has a Nearctic origin (Schöller 2023), but it has been spreading intensively in the last five decades and currently it can be considered as having a global distribution. It is a pest on sweet potato, soft berries, leucaena and other vegetables. This species was first found at Parque Urbano (Ponta Delgada), São Miguel Island, on 25.04.2022, using a sweeping net and later found in other locations (Lagoa, Vila Franca, São Roque).

### Diboliaoccultans (Koch, 1803) (Coleoptera, Chrysomelidae)

This small-bodied flea beetle (Alticinae) species is widespread in Europe, North Africa, Anatolia and the Caucasus. It occurs in Portugal and the Canary Islands (Warchalowski 2003). It is an oligophagous species and its larvae mine into the leaves of Lamiaceae, such as *Mentha* and *Prunella* spp. The species was first collected in the Azores from Pico Island, in 2015, but at that time, it was not formally identified (reported as morphospecies MF 1372 in Lhoumeau et al. (2024)). In 2024, a single specimen was collected by hand at Matela, Terceira island, on the 12.04.2024 and then several more specimens from the same location on the 08.07.2024, using a sweep net. On each occasion, the beetles were found on *Mentha*. An earlier record from Horta is found on iNaturalist (22.05.2022), but since the actual specimen is missing, this cannot be confirmed. Yet, photos and the fact that the beetle was recorded from *Mentha* make the report likely to be valid.

### Phyllotretaprocera (Redtenbacher, 1849) (Coleoptera, Chrysomelidae)

This uniformly coloured *Phyllotreta* species is 2-3 mm long and has slightly metallic body with a green hue and dark antennae. It is a circummediterranean species, occurring also in Central and Eastern Europe, Anatolia, eastern Africa and the Caucasus. Although it is distributed across Macaronesia (Cape Verde, Canary Islands and Madeira) (Erber 1986), this is its first record from the Azores. The first specimen was collected on Pico Island in 2019 using a SLAM trap (see MF1709 in Borges and Lhoumeau (2024b)), but has only been identified recently. Its host plants are *Reseda* spp. and Cruciferae. On the latter taxon, they rarely can act as pests.

### Phyllotretastriolata (Fabricius, 1803) (Coleoptera, Chrysomelidae)

The striped flea beetle is distributed throughout the Palaearctic Region and it has been introduced into eastern North America and South Africa. Its body is relatively small (1.8-2.2 mm), shiny black with wavy longitudinal yellow stripes on the elytra which are often present as two separated marks (Warchalowski 2003). It is a direct pest of Brassicaceae, but may also cause indirect damage by acting as a vector for plant diseases (Soroka and Grenkow 2013). It was collected from Pico Galhardo native forest plot in Terceira Island, in 2013 and later from the edge margin of Terra-Brava native forest plot, using SLAM traps. It was also found in Santa Maria Island. The specimens were recorded as morphospecies MF1246 for Terceira (see Borges et al. (2022c)) plus Santa Maria (Borges et al. 2024) and only recently identified to species level.

### Neoderelomuspiriformis (Hoffmann, 1938) (Coleoptera, Curculionidae)

The true weevil *Neoderelomuspiriformis* (Hoffmann, 1938) was recently recorded for Terceira Island by Lamelas-Lopez et al. (2023), but without mentioning the fact that this was, indeed, the first record for the Azorean Islands. Distribution data on this species can be accessed in GBIF (Borges and Lamelas-López 2023). This is an exotic species developing on Canary Island date palm *Phoenixcanariensis* hort. ex Chabaud (Friedman 2006) and not surprisingly was recorded in “Jardim Duque da Terceira”, a public garden characterised by the importation of exotic plants. The species is known from the Canary Islands (Izquierdo et al. 2001, Arechavaleta et al. 2010) and has been expanding to several other regions, including Greece (Kakiopoulos et al. 2022) and Israel (Friedman 2006).

### Chrysomyaalbiceps (Wiedemann, 1819) (Diptera, Calliphoridae)

**Figure 6.**
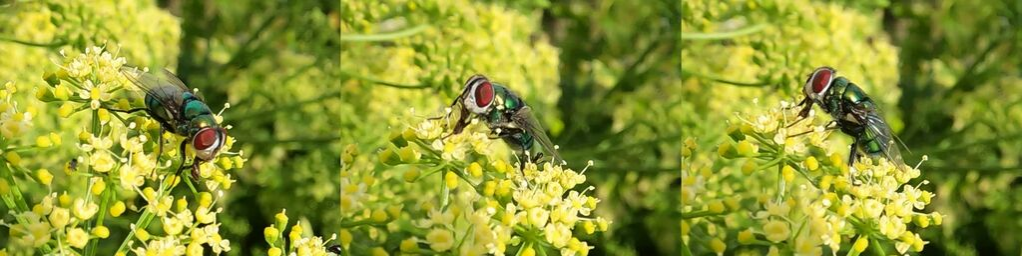
The blowfly *Chrysomyaalbiceps* Wiedemann, 1819 visiting *Petroselinumcrispum* (Mill.) Fuss (Apiales, Apiaceae). Photos by Zsófia Varga-Szilay.

*Chrysomyaalbiceps* (Wiedemann, 1819) is a blowfly originally from Africa, the Iberian Peninsula and the Middle East. However, the species is now found worldwide, with a broad distribution in Africa, South America, Southwest Asia, the Middle East and Central Europe (Laurence 1981). Furthermore, it has also colonised Madeira and the Canary Islands and future scenarios indicate potential spread into cooler regions, particularly in northern and eastern Europe (Prado e Castro et al. 2016, Hosni et al. 2022, Rodrigues-Filho et al. 2023). As a vector of diseases and pathogens, such as causing myiasis in humans and livestock, this fly is of concern in both medical and veterinary contexts (Sotiraki and Hall 2012). It feeds and breeds on faeces and carrion, making it significant from both sanitary and forensic perspectives. Additionally, it provides relevant ecosystem services, such as being a natural enemy of other dipteran larvae and pollination (Faria et al. 1999, Cook et al. 2020). In the Azores, *C.albiceps* was first reported on São Miguel based on human observation (Frey 1945, Borges et al. 2010). Here, we report for the first time the presence of this species in Terceira Island following the observation of an individual on *Petroselinumcrispum* (Mill.) Fuss (Apiales, Apiaceae) in a suburban home garden, in Biscoitos (Fig. 6).

### Pseudolynchiacanariensis (Macquart, 1839) (Diptera, Hippoboscidae)

The pigeon louse fly, *Pseudolynchiacanariensis* (Macquart, 1839), is an ectoparasite of birds, most commonly pigeons and other Columbiformes, although in laboratory setting, no strong host specificity was observed (Klei and Degiusti 1975). This fly is known to be a vector of *Haemoproteuscolumbae* Kruse, 1890, a blood parasite that infects pigeons and is known to cause mortality in chicks, while often being benign to adults (Markus and Oosthuizen 1972, Knutie et al. 2013, Cepeda et al. 2019). The individuals were collected in the vicinity of a small feral pigeon colony of *Columbaliviadomestica* Gmelin, 1789 situated at the top of a building in Angra do Heroísmo (Terceira Island) and recorded in photographs uploaded to iNaturalist, which led to a preliminary identification that was further confirmed through the use of taxonomic identification keys (Oboňa et al. 2022). This is the first finding of this species in the Azores. Considering that *P.canariensis* flies can use other Columbiformes species as hosts, it is necessary to carry out studies to verify if they are jumping hosts, as the Azores has its own native species of pigeon, the Common Wood-Pigeon *Columbapalumbus* Linnaeus, 1758.

### Hermetiaillucens (Linnaeus, 1758) (Diptera, Stratiomyidae)

The black soldier fly, *Hermetiaillucens* (Linnaeus, 1758), is native to the Neotropics, but it has spread across all continents during the last decades (Demetriou et al. 2022). This fly has a medium size (ranging from 12-20 mm), long antennae, presents dark body colour with metallic reflections and characteristic translucent areas on tergite 2 and has infuscated wings (Üstüner et al. 2003) (Fig. 7). Due to its morphology and behaviour, it is considered as a wasp-mimicking species. The black soldier fly larvae can eat diverse organic waste and have been used to feed pets, livestock, poultry and fish (Wang and Shelomi 2017). In recent years, many composting facilities using black soldier fly larvae were established in order to process organic waste and to produce animal food, with major benefits for local economies and the environment (Shelomi 2020). Additional benefits of this species include the control of house fly infestations and its use as a tool in forensic entomology (Furman et al. 1959, Lord et al. 1994). However, given the potential negative impacts of this species on local biodiversity and ecological processes, precautionary assessment should be considered in its management. The species was recently reported for the first time in the Azores after being observed in São Miguel Island and we collected several individuals from two localities in this Island (Boieiro and Borges 2024).

### Xanthandrusazorensis Frey, 1945 (Diptera, Syrphidae)

**Figure 8.**
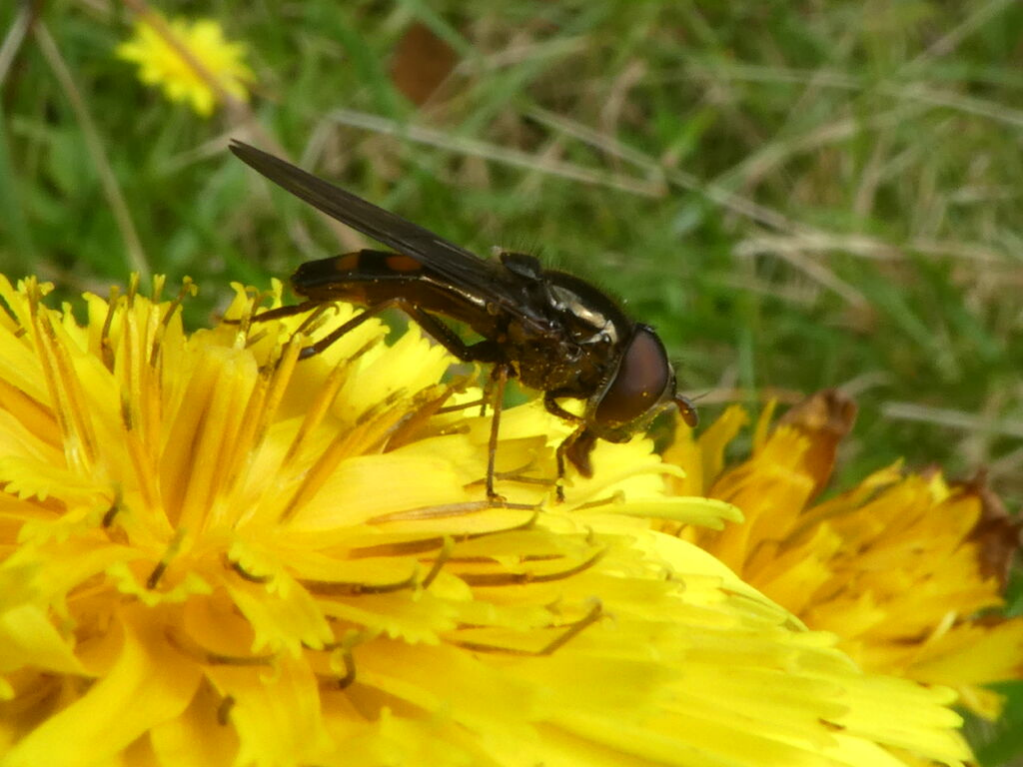
The endemic hoverfly *Xanthandrusazorensis* Frey, 1945 visiting a flower. Photo by Mário Boieiro.

The hoverfly *Xanthandrusazorensis* Frey, 1945 is an endemic species to the Azores Archipelago, already recorded in several islands (Borges et al. 2010, Jentzsch 2014, Borges et al. 2022a). This robust species is 9-10 mm long, has a dark body with abdominal markings and bluish-metallic lustre. It is very similar to the Madeiran endemic *Xanthandrusbabyssa* (Walker, 1849) and can be separated from its Azorean congener *X.comtus* (Harris, 1780) since the latter one presents a pair of yellowish spots on tergite 2 (T2) (Frey 1945, Rojo et al. 1997, Rego et al. 2022). The larvae of *Xanthandrus* species are known for their important role against several agricultural and forestry pests, including aphids and caterpillars, but there is no information concerning the *X.azorensis* diet. The adults of *X.azorensis* can be found collecting pollen and nectar from flowers of both native and exotic plants (Ferreira 2018, unpublished data) (Fig. 8). This Azorean endemic species is classified as Near Threatened (NT) in the IUCN Red List of Threatened Species (Nunes et al. 2022, Vujić et al. 2022). In recent years, the species was reported for the first time from Terceira Island, mostly in grey literature (Picanço et al. 2017, Ferreira 2018, Picanço 2018, Gabriel and Borges 2019). We have confirmed its occurrence on Terceira and found several individuals in different habitat-types (e.g. native forest, naturalised vegetation and semi-natural pastures) and occasionally found it in abundance. Future attention should be given to this species in order to understand the extent to which it can be used in biological control programmes in agricultural and forestry systems.

### Cryptoblabesgnidiella (Millière, 1867) (Lepidoptera, Pyralidae)

The honeydew moth *Cryptoblabesgnidiella* (Millière, 1867) is known for its association with various crops and its potential impacts as a pest, especially in vine cultivations. These moths are small, with a wingspan of about 12-20 mm. The fore-wings are generally brownish with darker markings, while the hind-wings are lighter, often greyish-white. The caterpillars are typically pale yellow to greenish with a reddish-brown head. They can reach up to 15 mm in length when fully grown. The damage caused by *C.gnidiella* can lead to reduced yields and economic losses for farmers (Dawidowicz and Rozwałka 2016). The moth is particularly problematic in vineyards, citrus groves and other fruit orchards (Keçeci 2021). Monitoring techniques, such as pheromone traps, are commonly used to detect and manage populations in agricultural settings (Ricciardi et al. 2021). The species was previously known from few sites in Pico, Terceira and São Miguel Islands (Borges et al. 2022a), but recent findings show that it is expanding its distribution and causing significant economic impacts, particularly in Biscoitos (Terceira Island).

### Chrysisignita (Linnaeus, 1758) (Hymenoptera, Chrysididae)

**Figure 9.**
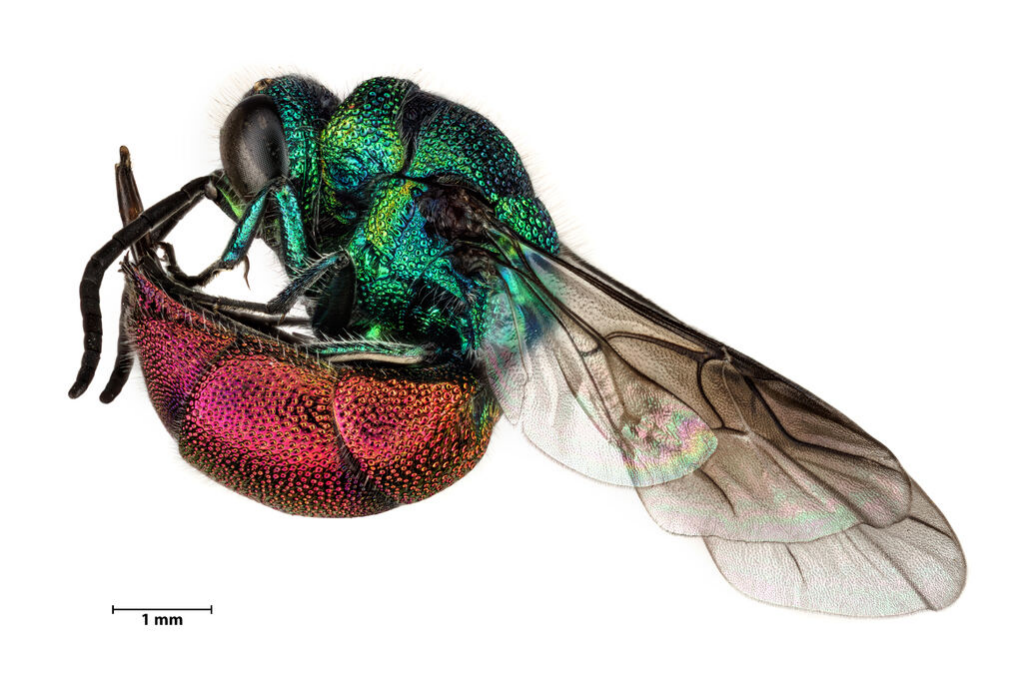
The ruby-tailed cuckoo wasp *Chrysisignita* (Linnaeus, 1758) in lateral view. Photo by Javier Torrent.

This ruby-tailed cuckoo wasp, *Chrysisignita* (Linnaeus, 1758), is 5-10 mm in size, the head and mesosoma are dorsally blue or violet and the abdomen has golden-red tergites and green or blue sternites (Fig. 9). This species can be separated from other congeners by its M-shaped or arcuate frontal carina, the vertex has white pubescence (occasionally brown in males), abdominal tergites T2 and T3 have coarse and regular punctuation and sharp apical teeth and sternite S2 has subrectangular black spots (Paukkunen et al. 2015). The species occurs in the Palaearctic Region with records from many European countries, but also from China, South Korea and Japan (Paukkunen et al. 2015). It is a cleptoparasitic wasp that, during larval development, uses the food items stored in its hosts' nests, which are generally potter wasps of the genus *Ancistrocerus* (Paukkunen et al. 2018). This is the first record of this species for São Jorge Island, but the species is known to occur on other Azorean islands (Borges et al. (2010), Borges et al. (2022a), see also https://azoresbioportal.uac.pt). A single specimen was found (on 30.08.2022) close to a window in an urban area (Calheta, São Jorge). In Terceira Island, Picanço et al. (2017) have reported a single specimen from an exotic forest and we recently observed three individuals near a native forest patch at Algar do Carvão.

### Ectemniuscephalotes (Olivier, 1792) (Hymenoptera, Crabronidae)

*Ectemniuscephalotes* (Olivier, 1792) is a large species of square-headed wasp, measuring approximately 15 mm in length, with large compound eyes and exhibiting the familiar black and yellow colouring on the abdomen. It is one of the more common large *Ectemnius* species, widely distributed across Europe, western Asia and North America (Bitsh and Leclercq 1993). *Ectemniuscephalotes* typically nests in dead wood, making mature forests its primary habitat. However, it is also found in rural and urban areas where dead trees and fallen or decaying trunks are present. This species preys on and paralyses medium-sized flies, which it stores in the cells it excavates in soft wood; several females often share a nest entrance. This species is reported for the first time in the Azores. We collected a female individual in a suburban garden in Biscoitos (Terceira Island). Previously, *Ectemniuslapidarius* (Panzer, 1804) was the only species from this genus reported in the Azores, based solely on historical data with no preserved specimens and without indication of the collection site (Borges et al. 2010).

### Dryocosmuskuriphilus Yasumatsu, 1951 (Hymenoptera, Cynipidae)

The Asian chestnut gall wasp *Dryocosmuskuriphilus* Yasumatsu, 1951 was recently observed for the first time on Terceira Island. This is an invasive species native to China that has spread to various parts of the world, causing significant damage to chestnut trees (*Castanea* spp.) (Grazioli and Santi 2008). The adult wasp is small, about 2.5 to 3.5 mm long, with a black body and yellowish legs. The larvae are white and are found inside galls on the chestnut tree (Avtzis et al. 2019). Not surprisingly, the species was found on Terceira associated with European chestnut (*C.sativa* Mill.) trees in the localities of Terra-Chã and Biscoitos. Infestation by *D.kuriphilus* can cause significant damage to chestnut trees, leading to reduced nut production, weakened trees and, in severe cases, tree death (Grazioli and Santi 2008, Avtzis et al. 2019). Monitoring and removal of infested plant material can help manage the spread of the pest. Although it is an impractical solution for large-scale application, pruning the galled shoots can also reduce infestation levels (Bernardo et al. 2013). One of the most effective biological control agents, the parasitoid wasp *Torymussinensis* (Linnaeus, 1758), has been successfully used in Japan and other countries (Nieves-Aldrey et al. 2019).

### Lasioglossumlativentre (Schenck, 1853) (Hymenoptera, Halictidae)

The furry-claspered furrow bee, *Lasioglossumlativentre* (Schenck, 1853), has a medium size (7–9 mm), roundish head, light brown stigma, characteristic sparse punctuation on tergite T1 and narrow bands of pale hair (on T2 and T3) (Amiet et al. 2001, Falk 2017, Weissmann et al. 2017). This species has a Western Palaearctic distribution, ranging from the Iberian Peninsula to Iran and was introduced in the Azores (Stöckl 1998, Weissmann et al. 2017). It is a solitary bee that visits mainly flowers of Asteraceae, but was also recorded visiting species of other plant families (e.g. Ericaceae, Ranunculaceae, Rosaceae and Salicaceae). This is the first record of this species for Terceira Island, but it has been previously reported from Faial and São Miguel Islands (Weissmann et al. (2017), see also https://azoresbioportal.uac.pt). On Terceira, the species was found in several locations (Boieiro and Borges 2024), mainly in pastures, during the summer of 2023.

### Anthidiummanicatum (Linnaeus, 1758) (Hymenoptera, Megachilidae)

The wool carder bee, *Anthidiummanicatum* (Linnaeus, 1758), is a solitary bee native to and widely distributed across Europe, Asia and North Africa, but it is also present in South America, New Zealand and the Canary Islands (Soper and Beggs 2013) as the species is rapidly expanding its range worldwide (Gibbs and Sheffield 2009). *Anthidiummanicatum* was first recorded from São Miguel Island, in 1857 and subsequently reported from Faial (Weissmann et al. 2017). More recently, the species was observed on São Jorge (August 2021), Flores (25.07.2023) and Terceira Islands (31.07.2023). In Terceira Island, the species was observed on the flowers of the hairy-leaved *Coleusbarbatus* (Andrews) Benth (Lamiales, Lamiaceae) in the Campus of the University of the Azores and two individuals were recently collected on *Lotuspedunculatus* Cav. (Fabaceae) at the industrial complex of Angra do Heroísmo. This species often visits plants with hairy leaves, from which females collect soft hairs to be used in their nests. Males are highly territorial and aggressive towards conspecifics (Pechuman 1967), as well as towards native pollinating insects (Taggar et al. 2021) and strongly compete for resources within their territories.

### Ancistrocerusgazella (Panzer, 1798) (Hymenoptera, Vespidae)

The European potter wasp, *Ancistrocerusgazella* (Panzer, 1798), is a medium-size insect (7.5-9 mm) that feeds on caterpillars during the larval stage, while adults eat nectar and aphid honeydew. This species occurs in mainland Europe, North Africa and the Middle East and was introduced into North America and New Zealand (Harris 1994, Gereys 2016). This is the first record of this species for Terceira Island, but it is also known from Faial and Pico Islands (Borges et al. (2010), Borges et al. (2022a), see also https://azoresbioportal.uac.pt). In Terceira, the species was collected from several locations during the summer of 2023, mostly in semi-natural and intensive pastures. *Ancistrocerusgazella* is very similar to its congener *A.parietum* (Linnaeus, 1758), also known to occur in the Azores, but this latter species has a deep V-shaped medial incision in the transverse carina of tergite T1 (Buck et al. 2008, Gereys 2016).

## Discussion

In the Azores, the current situation matches the general concern on the consequences of the increased introduction of exotic species on islands (Pimentel 2011, Silva-Rocha et al. 2018, Zenni et al. 2021). Many exotic species were accidentally introduced in the Azores during this century, some of which are threatening native biodiversity and ecological processes and have led to significant economic costs (e.g. Borges and Myles (2007), Silva et al. (2008), Vieira (2008), Guerreiro et al. (2014)). Our recent findings also raise a great concern since several species here reported are potentially harmful, considering their effects on agriculture, forestry and veterinary. For example, the Persea mite (*Oligonychusperseae*), the desert locust (*Schistocercagregaria*), the eucalyptus longhorned borer (*Phoracantharecurva*), the Asian chestnut gall wasp (*Dryocosmuskuriphilus*), the honeydew moth (*Cryptoblabesgnidiella*) and the flea beetles *Phyllotretaprocera* and *P.striolata*, are often reported as important agricultural and/or forestry pests due to their severe impacts on diverse yields (Dawidowicz and Rozwałka 2016, Keçeci 2021, EPPO 2024, Torres et al. 2024). The honeydew moth is a generalist species feeding on a wide variety of plants, mostly commercial crops, including maize (*Zeamays* L.) and various species of *Citrus*, *Diospyros*, *Malus*, *Persea*, *Prunus*, *Punica*, *Pyrus* and *Vitis* (Lucchi et al. 2019, Velez-Gavilan 2023). Despite host range and honeydew moth preferences being insufficiently known in Azores, the rural landscape with mixed, diverse and locally abundant cultures may favour the rapid spread of this species. Thus, the production of several goods (e.g. maize, grapes/wine, apples) will likely suffer a serious reduction in the forthcoming years with potential negative impacts on the local economies. The impacts of other species, such as the eucalyptus longhorned borer and the Persea mite, are expected to be more local and easier to manage since these are phytophagous and specialists, attacking fewer host plants in smaller areas. Nevertheless, at this stage, the economic consequences of these introductions are still difficult to predict since some species may not be able to establish due to environmental filtering and biotic resistance, while others may rapidly invade the suitable areas in the Archipelago, benefitting from human assisted dispersal, the high availability of human modified habitats and facilitation provided by established exotics and native generalists. For instance, the unsuccessful case of the deliberated introduction of *Harmoniaaxyridis* (Pallas, 1773) in the Azores showed that species establishment is a complex process driven by invasiveness, invasibility and interactions with the local fauna and flora (Honek et al. 2017, Soares et al. 2017, Soares et al. 2018).

The unique Azorean biodiversity and native ecological processes are also threatened by the constant arrival and spread of exotic species, which are now a major fraction of the terrestrial arthropod species richness of the Archipelago (Borges et al. 2020, Borges et al. 2022a). Overall, 951 out of the 2436 terrestrial arthropod species and subspecies reported to the Azores (39%) are considered introduced. Some exotic predators, like the spiders *D.umbratilis*, *P.audax* and *Z.spinimana*, may drive changes in the abundance and composition of co-occurring prey and predator communities, potentially outcompeting native species, as has been reported in other archipelagos (Cardoso et al. 2010, Wetterer and Espadaler 2010). Additionally, introduced flower visitors, like the honeybee and *A.manicatum*, can deplete local nectar and pollen resources and interfere with flower visitation by native pollinators through aggressive territorial behaviour. Thus, exploitation and interference competition for floral resources between exotic and native pollinators may lead to declines in species abundance and richness and also influence the reproductive success of native plants (Soper and Beggs 2013, Valido et al. 2014, Valido et al. 2019, Taggar et al. 2021). The introduction of parasites and diseases, together with their hosts, is also a major issue despite being often neglected. The consequences of these co-introductions on native terrestrial arthropod biodiversity usually remain unnoticed, but can be tragic. For instance, the extinction of the Madeira endemic large white butterfly (*Pieriswollastoni* (Butler, 1886)) is attributed to the introduction of infected individuals of the congener *P.rapae* Linnaeus, 1758 which brought with them both a virus and a parasitoid to which the endemic species was highly vulnerable (Gardiner 2003). The recent finding of the pigeon louse fly (*P.canariensis*) in the Azores, a vector of the blood parasite *Haemoproteuscolumbae*, in a pigeon colony of *C.livia* raises some concern on the consequences of the potential host shift to the native *C.palumbus* as the disease can be fatal to chicks (Cepeda et al. 2019). In the Azores, most introduced species have only been reported from human modified habitats (e.g. agricultural and urban areas, forest plantations, pastures) and there is some evidence that they may not establish in the remaining native forest fragments which are located at higher altitudes with harsher climate and offer significant biotic resistance (Cardoso et al. 2009, Tsafack et al. 2021). Nevertheless, several introduced species, like the flea beetle *P.striolata*, managed to colonise the native forest, being important to monitor their spread, the changes in population size and the impacts on native species.

The globalisation of trade and transportation has reduced the effective isolation of oceanic islands and exposed their native biota and human societies to a significant pressure from alien species introductions. The arrival and spread of introduced species on islands are usually mediated by inadvertent human-assisted dispersal as illustrated by the rapid dissemination of the exotic bush cricket *T.cicindeloides* across Azorean islands. This cricket was found for the first time in São Miguel Island, near the airport (Borges et al. 2013) and was later reported from Terceira and Santa Maria Islands also from the vicinities of the local airports from where it has been spreading to the neighbouring suburban and urban areas (Borges et al. 2018, this study). In order to prevent new introductions and mitigate their socio-economic consequences, the Azorean governmental organisations need to implement a coordinated strategy and concerted efforts, supported by scientific knowledge, specific legislation and funding (Aguin-Pombo et al. 2010, Hulme 2015). It is fundamental to identify the main vectors and pathways of introductions in the Azorean islands and improve quarantine measures and product inspections to prevent new species arrivals. In addition, it is crucial to set up a monitoring programme for the early detection of alien species in the Azores, targeting at least the most frequent introduction pathways (airports and harbours), to allow a rapid response to prevent species establishment and spread. A specific programme for the early detection of the invasive Asian hornet (*Vespavelutina* Lepeletier, 1836) is ongoing, but to our knowledge, no other initiatives are being carried out. Potentially harmful introduced species (including several reported here) also need to be promptly identified and targeted for specific monitoring programmes that need to take into account the particular characteristics of their biology and impacts and should be aimed to timely assess the need for the adoption of control/eradication actions. All of these activities must be accompanied by education and awareness campaigns aimed specifically at professionals in the agricultural, forestry, nature conservation and veterinary sectors, but also to the general population and decision-makers.

Managing the ongoing increase in species introductions and biological invasions is undoubtedly one of the main challenges for insular territories worldwide and the success in addressing this issue will largely depend on the commitment to implement specific legislation, improve quarantine measures and product inspections, invest in monitoring and control actions and carry out work collaboratively with informed decision-makers, stakeholders and the public.

## Figures and Tables

**Figure 1a.**
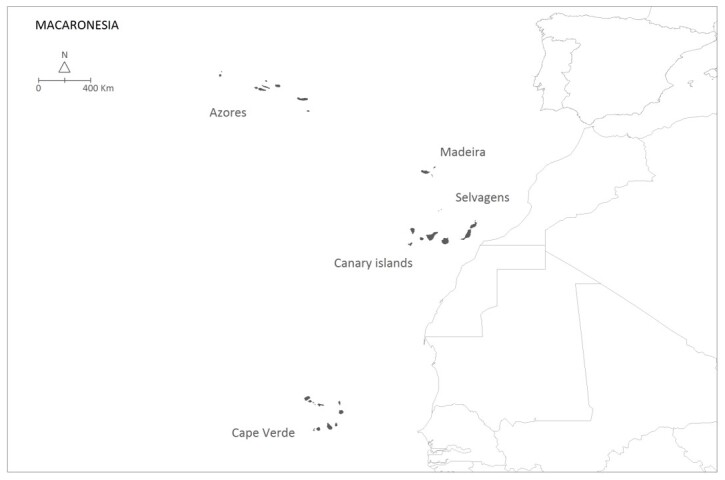
Macaronesia

**Figure 1b.**
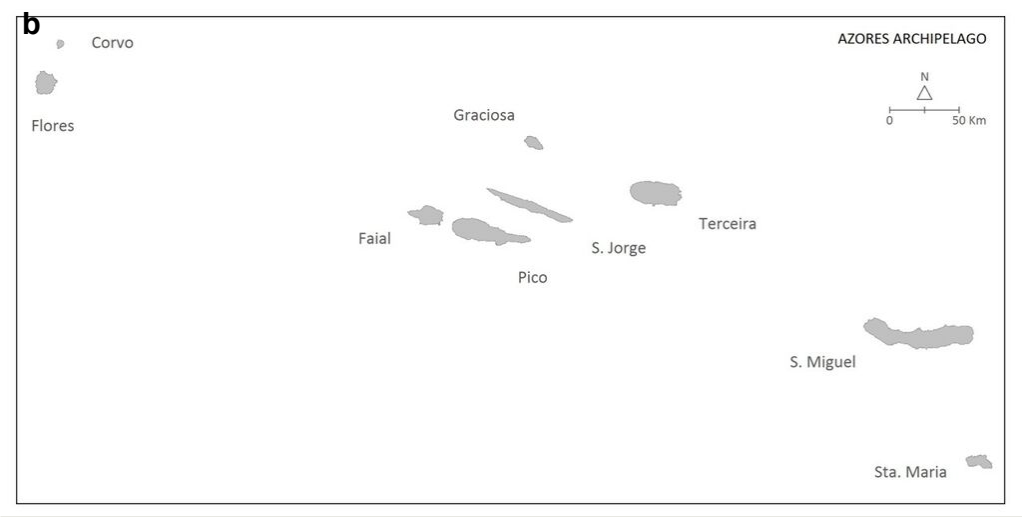
The Archipelago of the Azores

**Figure 4a.**
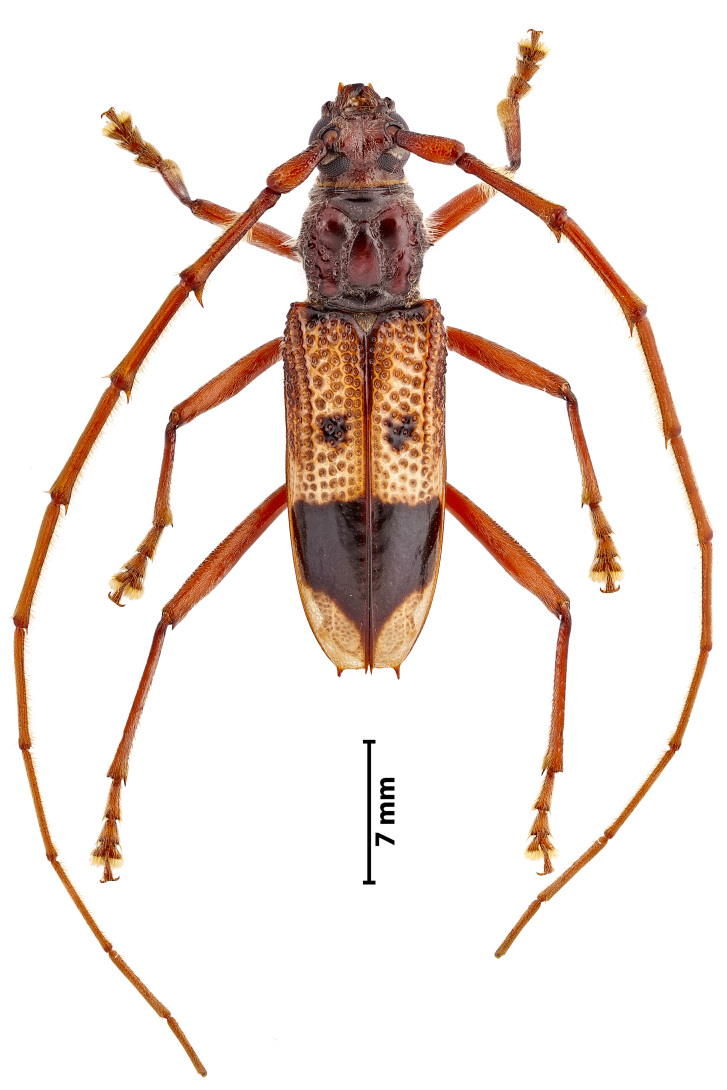
Dorsal view

**Figure 4b.**
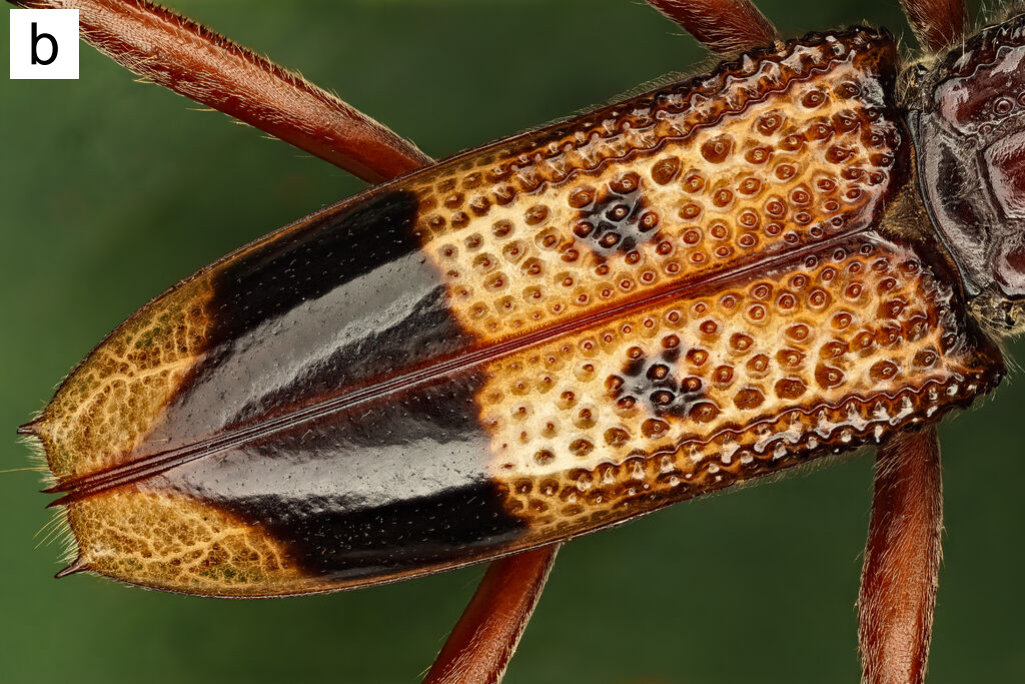
Details of the elytra

**Figure 5a.**
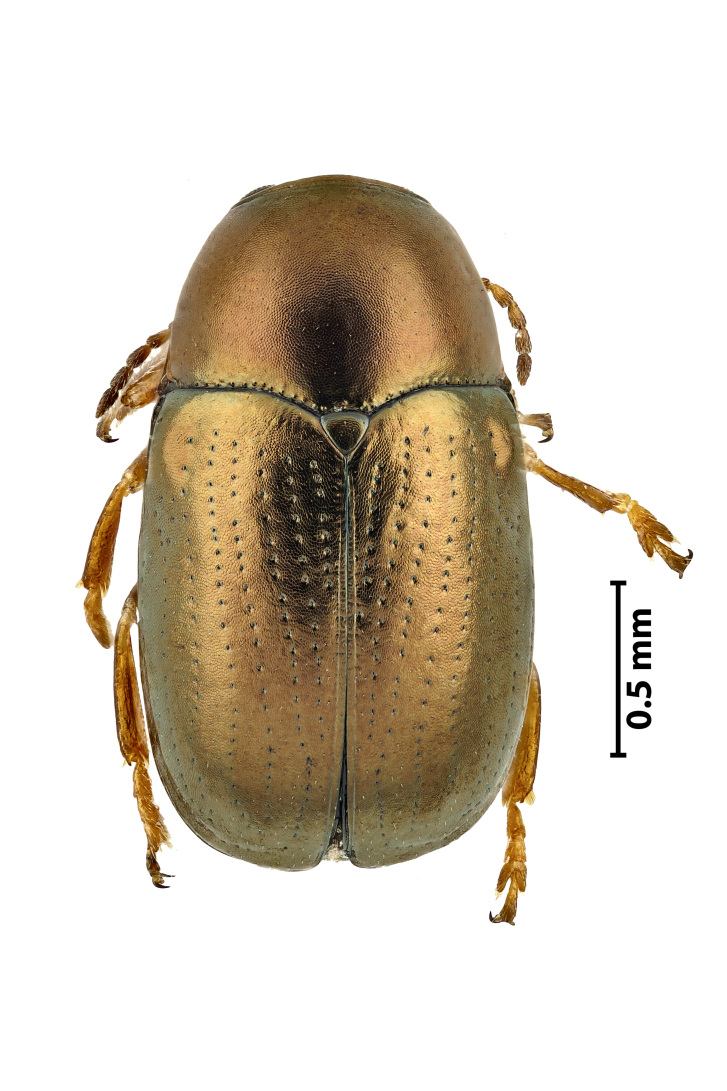
Dorsal view

**Figure 5b.**
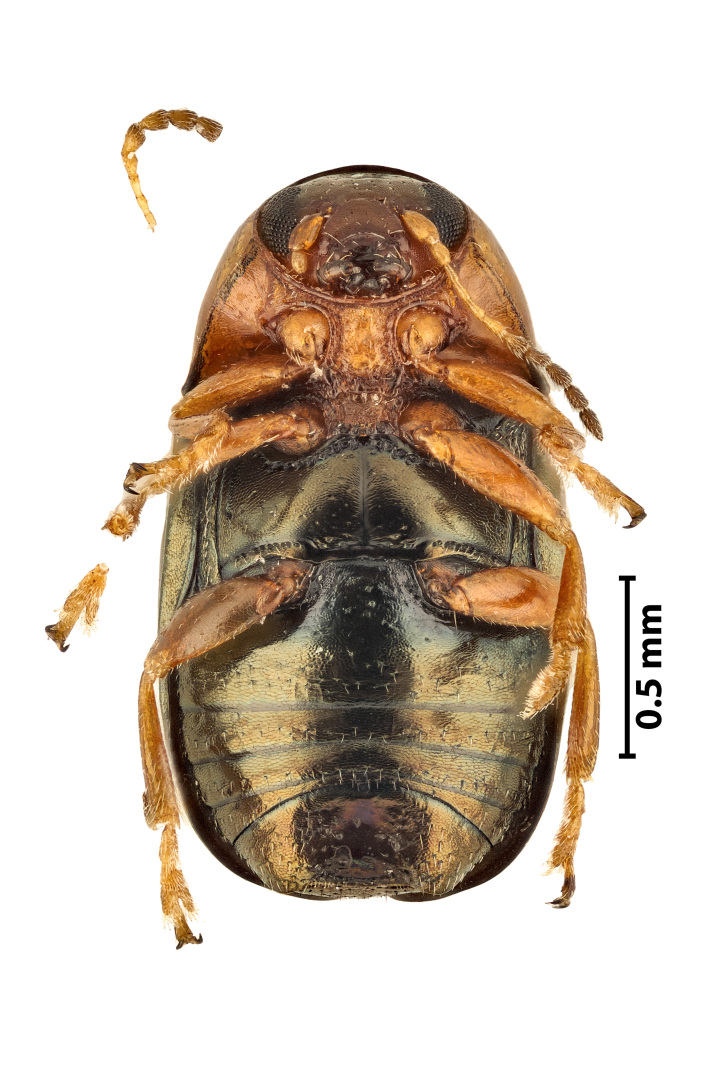
Ventral view

**Figure 7a.**
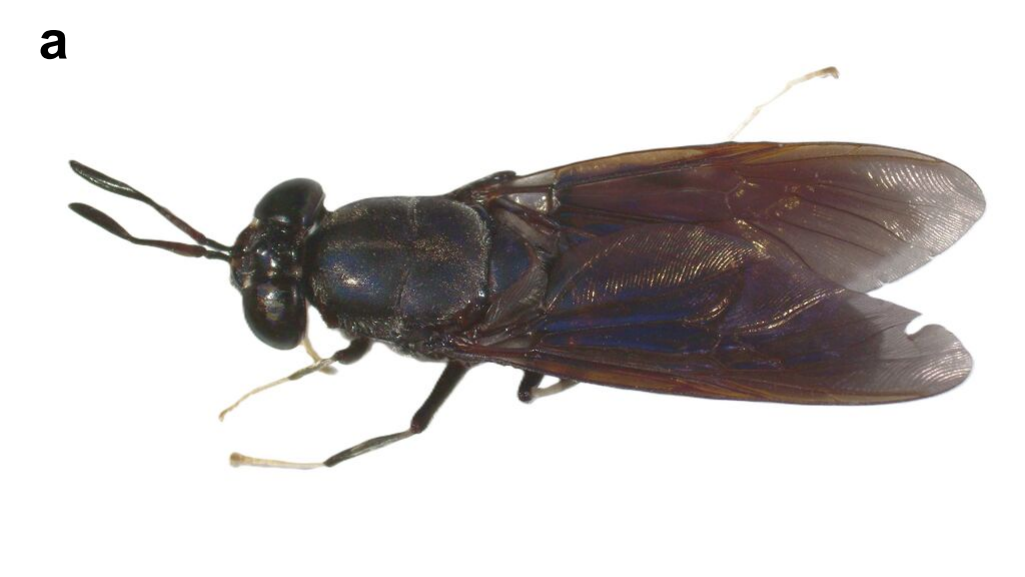
Dorsal view

**Figure 7b.**
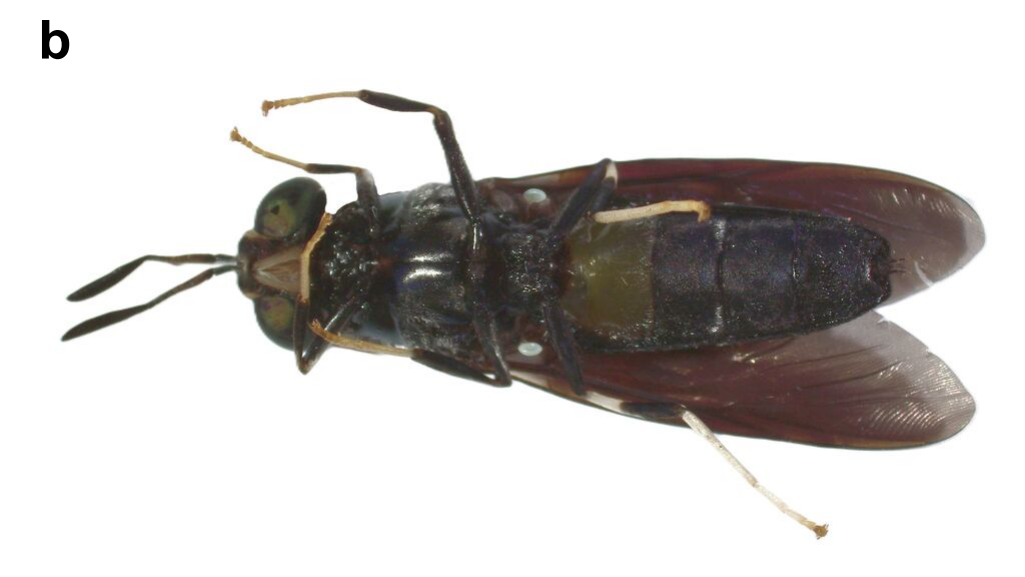
Ventral view

**Table 1.** List of the terrestrial arthropod species reported as new to the Azores or to specific Azorean islands with indication of their taxonomic group and distribution status (END - endemic; NAT - native; INT - introduced). The novel distribution findings (X) at island level are recorded jointly with previous records (o) following Borges et al. (2022a) (see also https://azoresbioportal.uac.pt/). The names of Azorean islands are abbreviated as follows: Corvo (COR), Flores (FLO), Faial (FAI), Pico (PIC), Graciosa (GRA), São Jorge (SJG), Terceira (TER), São Miguel (SMG) and Santa Maria (SMR).

Species	Taxonomic group	Distribution status	Distribution in Azorean Islands								
			COR	FLO	FAI	PIC	GRA	SJG	TER	SMG	SMR
*Oligonychusperseae* Tuttle, Baker & Abbatiello, 1976	Acarina, Tetranychidae	INT							X		
*Textrixpinicola* Simon, 1875	Araneae, Agenelidae	INT		X							
*Agynetarugosa* Wunderlich, 1992	Araneae, Linyphiidae	END			o			o	X	o	
*Phidippusaudax* (Hentz, 1845)	Araneae, Salticidae	INT							o		
*Dipoenaumbratilis* (Simon, 1873)	Araneae, Theridiidae	INT			o	X					
*Pholcommagibbum* (Westring, 1851)	Araneae, Theridiidae	INT		X							
*Zoropsisspinimana* (Dufour, 1820)	Araneae, Zoropsidae	INT		X	o	o			o		
*Schistocercagregaria* (Forsskål, 1775)	Orthoptera, Acrididae	vagrant							X		
*Trigonidiumcicindeloides* Rambur, 1838	Orthoptera, Trigonidiidae	INT							o	o	X
*Nabiscapsiformis* Germar, 1838	Hemiptera, Nabidae	INT		o	o				X	o	o
*Phoracantharecurva* Newman, 1840	Coleoptera, Cerambycidae	INT							X		
*Diachusauratus* Fabricius, 1801	Coleoptera, Chrysomelidae	INT								X	
*Diboliaoccultans* (Koch, 1803)	Coleoptera, Chrysomelidae	INT			?	X			X		
*Phyllotretaprocera* (Redtenbacher, 1849)	Coleoptera, Chrysomelidae	INT?				X					
*Phyllotretastriolata* (Fabricius, 1803)	Coleoptera, Chrysomelidae	INT							X		X
*Neoderelomuspiriformis* (Hoffmann, 1938)	Coleoptera, Curculionidae	INT							o		
*Chrysomyaalbiceps* (Wiedemann, 1819)	Diptera, Calliphoridae	INT							X	o	
*Pseudolynchiacanariensis* (Macquart, 1839)	Diptera, Hippoboscidae	INT							X		
*Hermetiaillucens* (Linnaeus, 1758)	Diptera, Stratiomyidae	INT								X	
*Xanthandrusazorensis* Frey, 1945	Diptera, Syrphidae	END			o	o		o	o	o	
*Cryptoblabesgnidiella* (Millière, 1867)	Lepidoptera, Pyralidae	INT				o			o	o	
*Chrysisignita* (Linnaeus, 1758)	Hymenoptera, Chrysididae	NAT		o	o	o		X	o	o	o
*Ectemniuscephalotes* (Olivier, 1792)	Hymenoptera, Crabronidae	INT?							X		
*Dryocosmuskuriphilus* Yasumatsu, 1951	Hymenoptera, Cynipidae	INT							X		
*Lasioglossumlativentre* (Schenck, 1853)	Hymenoptera, Halictidae	INT?			o				X	o	
*Anthidiummanicatum* (Linnaeus, 1758)	Hymenoptera, Megachilidae	INT		X	o	o		o	X	o	
*Ancistrocerusgazella* (Panzer, 1798)	Hymenoptera, Vespidae	NAT			o	o			X		
